# Progress in obstetrics or the road to the Promised Land

**Published:** 2008-02-25

**Authors:** R Vlădăreanu

**Affiliations:** University of Medicine and Pharmacy Carol Davila, Departament of Obstetrics and Gynecology, University Emergency Hospital Elias, BucharestRomania

## Abstract

The exceptional progress in obstetrics, especially in the second half of the 20th century, creates a series of dilemmas, without having yet the answer to old, basic questions, such as premature delivery and prevention of preeclampsia. Sexual liberty and the delay of the first pregnancy are responsible for the drop in natality rate and the increasing demand for assisted reproductive technologies. Their success is marked, though, by a drastic increase in the multiple birth rate and its complications. The advancing maternal age brings along a number of cardiovascular pathologies that endanger not only the present pregnancy, but also the long distance health prognosis. Plus, there is a marked risk for foetal chromosomal anomalies, which explains the need and the importance of prenatal diagnosis. Probably the newest branch in materno–foetal medicine, it offers many answers and solutions, but there are still a lot of question marks. In conclusion, the progress is necessary, is expected, but it should always be filtered by time and experience.

Obstetrics, a science born at the same time with human kind, having been crucially assimilated within its development and progress, has slowly evolved through thousands of years and today, we like to think that we have reached a point that comes close to the apex of our pyramid of knowledge. A peak where there are no longer uncertainties, where we do not longer have materno–foetal mortality and morbidity statistics, where human reproduction is a risk free phenomenon. Reality unfortunately, begs to differ.

The road of obstetrics may begin with Hippocrates, the one who assumed that birth is triggered by the foetus who ‘becomes agitated and thus breaks the membranes’, may continue with Leonardo da Vinci who, during the Renaissance, struggles to imagine the intrauterine development of the foetus, noting that it ‘has a daily growth inside the womb that surpasses its growth after birth’.

All great humanists, regardless of the period in time in which they lived and thought, were preoccupied by the decryption of the intrauterine growth mystery. What the twentieth century and especially its second half brings new, is the concern to treat and to prevent complications associated to pregnancy and birth. The development of molecular biology and genetics, the understanding of physiological mechanisms and their pathological alterations, the spectacular progress of computer technology with its potential applications in medicine, have cleared up for good the mystery of emergence of the human race, which was transformed from a myth into a science with numerous extensions and overspecializations, a science where the assistance of birth, a hundred years ago the basis of the trade, is now of secondary importance. Thus, at this moment, it is more appropriately to replace the term ‘obstetrics’ with the one ‘maternofoetal medicine’, more comprehensive and including both participants of this inseparable partnership[Fig 1].

**Fig 1:**
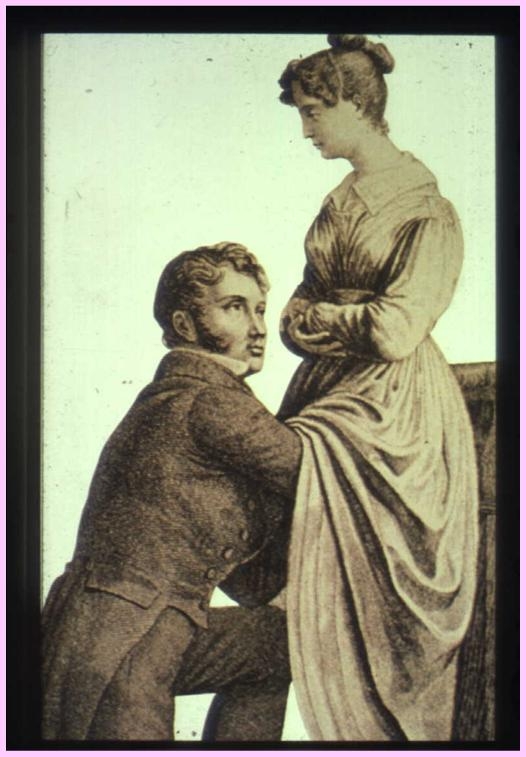
Obstetrical consult in the 19th century

The peak of the pyramid is still far. Even though outnumbered hypotheses were issued, many theories were made, a great deal of studies were elaborated, thousands of articles and papers were written, the statistical numbers are tough and show that the percentage of premature births is still high, almost unchanged in the last decades and the impairments caused by them constitute a great social, moral and economic burden, even in the developed countries with high medical standards. To be more precise in this matter, it must be noted that in 2005 in the United States, 12.7% of all births were premature, understanding by this a gestational age of less than 37 finished weeks or 259 days. 2.03% happened before 37 weeks while 9.1% happened between 34 and 36 weeks [1]. This aspect reflects an increase of 20% in the percentage of prematurity in comparison with 1990. How can this phenomenon be explained within the context of scientific and financial efforts to detect high risk of prematurity in pregnant women and to obtain pharmacological classes with high potential of reducing uterine contractility. The answer lies in the increased incidence of gemellar, triple and even more that triple pregnancies. The same statistical data reveal that between 1996 and 2002 multiple pregnancy rate rose up to 20 to 33% in a 1000 live births (a medium of 31/1000 for gemellar pregnancies) [2]. In comparison to one foetus pregnancies, with an overall preterm birth risk of approximately 10%, gemellar pregnancies have a 50% risk of being delivered before 37 weeks, and in triple pregnancy cases, this risk goes up to 90%. But as it is well known, preterm delivery implies low weight at birth, frequent incomplete development of the respiratory system, with consequent distress of variable severity, immaturity of the digestive tract, neurological frailty and possibly long term impairment. Thereby, almost 50% of neonatal deaths happen to those 1,5 newborns who weigh less than 1500 grams [3].  Between 1500 grams and 2500 grams there is also an addition of 20% mortality rate so that the relationship between gestational age and morbidity and mortality is clear and decreases exponentially as normal term comes nearer and nearer. Following the same line of thought, the survivors of a preterm birth at 26 weeks have a 60% incidence of impairment and disability, compared to the 30% rate for those born at 31 weeks [4]. Neonatal intensive care therapy has known a great progress and has increased survival chances of premature babies, although far from being an absolute resource as the above statistics have presented.

Multiple pregnancy constitutes a true epidemic of the last decade, having been undoubtedly a result of clinical success of assisted reproduction techniques. In natural conditions, the incidence of twin pregnancies is 1 to 80 and that of triple pregnancies is 1 to 8000, according to Hellin's law. In practice though, in the United States, in 2002 twin pregnancies made up 4% of all births and triple pregnancies, 3% [5]. The increased number of triple and more than triple pregnancies is especially evident, but there is hope of reducing this phenomenon in the following years due to the control of the number of embryos that can be transferred within a cycle of in vitro fertilization. This was a logical measure that the authorized medical world has taken to decrease complications linked with multiple pregnancies and it is more strictly applied in European countries.

A sensible question is why there is an increased appeal to the assisted reproduction services. The way of life in particular of population in developed countries, emancipation of women, sexual liberation, effective hormonal contraception, planned delay of maternity in order to favour professional success, imbalance in the institution of family, all these have contributed to the decrease of natality in particular by the aging of gametes and the rise in endometriosis and pelvic inflammatory disease incidence. It is difficult to asses the prevalence of infertility prior to the sixties, when it was regarded as a sporadic infliction. Though we know that in 1982, 11% of female American population that sought to become pregnant had fecundity issues, in 2002 this percentage rose to 15% [6]. According to a study made by WHO on 8500 infertile couples, in 37% of them the cause was feminine and only 8% was masculine. In 35% of cases the aetiology was mixed, feminine and masculine [7].

The advanced maternal age may also generate other complications. First, women over 40 years of age tend to associate a series of pathologies like gestational diabetes, obesity, chronic hypertension, especially renal hypertension. The incidence of preeclampsia in this category of women is high, in particular if it is their first pregnancy. Maternal age represents an independent significant risk factor for the development of preeclampsia (RR 1.96, 95% CI 1.34–2.87) [8].

Although it is one of the first complications of pregnancy that has been isolated, identified and thoroughly described by obstetrics classics, in the age of genetic diagnosis in maternal blood, in utero therapy and stem cells, preeclampsia still constitutes an unresolved problem, a major source of maternal mortality and morbidity for which there are yet few efficient therapeutic resources, affecting between 10 to 20% of all pregnancies. Namely a ‘disease of theories’, which illustrates the frailty of the ground on which its physiopathological vision has been built, having as a result a poor efficiency of treatment, its definition criteria have little been changed since its first description.  As the complexity of this disease is revealed step by step, the list of biological alterations that accompany preeclampsia grows accordingly. This does not seem to improve significantly the prognosis of these pregnancies but merely complicates the specialists' goal. In long term, a women who has developed early preeclampsia, has a high risk of cardiovascular disease and by that understanding hypertension, cardiac ischemic disease, stroke and cardiac death.

This relative risk varies between 3.5 and 1.5, depending on studied population and criteria of definition [9, 10]. These studies show that women with preeclampsia history have an alteration of endothelial function and a vasodilation distanced to their pregnancy. Vascular change is accompanied also by insulin resistance, sympathetic and proinflammatory hyperactivity, alteration of the lipidic profile, all these representing elements of metabolic syndrome which is responsible of changing long term prognosis.

It is not clear to what extent these metabolic changes can be lowered or even be avoided completely by an effective therapy of the hypertension episode in pregnancy. It is well known that the origin of these changes lies in the early stage of pregnancy, in the period of trophoblastic invasion, while consequences appear much later. That's why, efforts are aimed at the prediction and prevention of this disease. A good screening test should be simple, certain, quick, cheap and reproducible and should allow medical intervention in order to prevent the disease or at least improve its prognosis.

The first predictive test used was the ‘roll–over test’, which, although efficient, was proven to be outdated. Serum measurements of angiogenic factors such as VEGF or PIGF (placental growth factor), angiogenic proteins, like soluble endogline (sEng) or fragments of VEGF type 1 receptor (s–Flt1), also known as fms–like tyrosine–kinase, prove to be more useful though hardly accessible. Thus, in the serum of women at risk of preeclampsia there is an increased ratio between s–Flt1 and sEng and a decreased ratio between VEGF and PlGF, which determine a diffuse endothelial lesion, with alteration in the capillary permeability [11]. These changes happen months before clinical manifestations. Same changes can be observed also in urine.

Uterine artery Doppler ultrasonography is one of the most known prediction techniques. Through its simplicity, it has also been established as a screening technique although its flaws are many, having great inter–observer variations and not being considered appropriate for a screening test. Even with these in mind, a met analysis of 43 studies, which included 42000 women, revealed that a positive Doppler test increases a woman's risk from 2,5% (baseline risk in general population) to 8 – 15%, while a negative test decreases this risk to 1,5% [12]. Theoretically at least, recognition of 90% of women who will develop early preeclampsia is made possible.

Similarly, although hyperuricaemia is frequent in preeclamptic women and there was a lasting idea that uric acid levels grow a few weeks before tensional values grow as well, a series of recent studies have proven that this test does not have a predictive value and therefore can not be used in screening [14].

In these conditions, clinical routine evaluation, with systematic measurement of arterial pressure and look out for preeclampsia signs, has a predictive value similar to the most costly and newest diagnostic tests. That is, we are exactly where we left off.

Another controversial area is the prenatal diagnosis, a completely new science, developed at the same time with ultrasonography and genetics. Clearly, it is the domain with the greatest progress and hopes for the future. If 30 years ago ultrasound served generally to confirm the position of foetus, its cardiac activity and at most, the diagnosis of brutish structural anomalies, now we track down discrete facial dimorphisms and the lack of corpus callosum formation. Technical progress has introduced the spirit of competition, imposed the re–evaluation of fine anatomy and embryology data with continuously up–to–date and new information in the field of biology and genetics. In this way, there has been an evolution to the point of prenatal detection of some pathologies that don't even have a known prognosis and there are not few the situations when we encounter couples who ask pregnancy termination because the foetus has an isolated great cisterna magna or a partial corpus callosum agenesis. There is also the reverse of the coin. The numerous serological and ultrasound tests confuse doctor and patient alike, the patient refusing thus any additional investigation guided by her faith and optimism. Amniocentesis, umbilical cord centesis, trophoblastic biopsy can constitute effective diagnosis means but at the same time as many risks for the evolution of pregnancy.

Following the diagnosis, an in utero therapy has been developed. Starting with Harrison who in the eighties performed the first intervention of this kind for a foetus with diaphragmatic hernia, there is a shift to a more conservative approach. Of course that the defect may be repaired before birth, but this does not improve too much the prognosis of the foetus who is consequently exposed to prematurity. Thus, at this moment, only minimal repair surgeries are practiced, by means of foetoscopy or only under ultrasound guidance, in cases of low urinary tract obstruction, unilateral pleural effusion, diaphragmatic hernia or isolated ventriculomegaly.[Fig 2]

**Fig 2:**
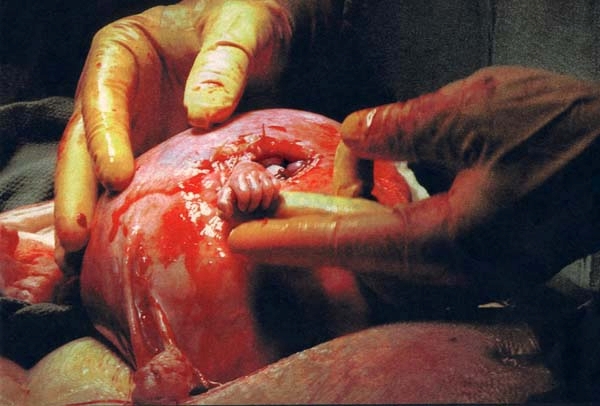
in utero surgery

Still, matters are far from ending at this point. Prenatal diagnosis in the future years will require the simple drawing of maternal blood in which foetal cells and foetal DNA will be isolated and used for all necessary tests. At least, that's what we would like to believe. We do not know yet to what extent this will simplify matters or on the contrary become another gate to unknown realms, but we owe belief and support to progress, filtering though the discoveries through our past experience and through the test of time.
